# Correlation between morphokinetic parameters and standard morphological assessment: what can we predict from early embryo development? A time-lapse-based experiment with 2085 blastocysts

**DOI:** 10.5935/1518-0557.20190088

**Published:** 2020

**Authors:** Catherine Jacobs, Mariana Nicolielo, Renata Erberelli, Fabiana Mendez, Marina Fanelli, Livia Cremonesi, Beatriz Aiello, Aline R. Lorenzon

**Affiliations:** 1 Embryology Department – Huntington Medicina Reprodutiva, São Paulo, SP, Brazil; 2 Scientific Coordinator – Huntington Medicina Reprodutiva, São Paulo, SP, Brazil

**Keywords:** time-lapse system, morphokinetics, morphology, blastocyst

## Abstract

**Objectives:**

To evaluate the association between morphology grading and morphokinetic parameters in blastocyst stage embryos cultured in a time-lapse system.

**Methods:**

This retrospective cohort study included patients offered fertility treatment with autologous oocytes in our clinic between October 2017 and May 2019 using a time-lapse system. The embryos were morphologically graded according to the criteria developed by Gardner and Schoolcraft and their morphokinetic parameters were recorded.

**Results:**

Our results indicated that the time of pronuclei fading (tPNf), time to cleavage into two (t2), four (t4), and eight (t8) cells, and time to start of blastulation (tB) were significantly different according to the morphological quality of the blastocysts formed. In the early development stage, tPNf, t2 and t4 differed between good (AA, AB, BA, BB) and poor (CC) quality potential blastocysts. The 8-cell stage time separated embryos graded as AA blastocysts in terms of morphology from embryos graded as BB. Earlier tB correlated with higher quality embryos (AA, AB, BA).

**Conclusion:**

Our results showed that the first kinetic parameters (tPNf, t2, and t4) distinguished top-graded from low-graded blastocysts. Between top-graded blastocysts, t8 separated BB blastocysts from AA blastocysts. And finally, tB also told apart BB blastocysts from AA, AB, and BA blastocysts. These time-related parameters may be applied even in centers without time-lapse systems.

## INTRODUCTION

In assisted reproductive technology (ART), morphological criteria are the basis for embryo development assessment. Although embryo categorization has become more robust, the dynamic nature of embryo development means they can change grading within a matter of hours (Gardner & Balaban, 2016). Embryo selection may be performed via invasive or non-invasive technologies. Non-invasive strategies include embryo morphology, time-lapse monitoring, metabolomics, and proteomic profiles, while invasive techniques involve embryo biopsy for genetic and/or chromosomal testing (Zaninovic *et al*., 2017).

Blastocysts are usually graded for morphology based on the criteria developed by Gardner and Schoolcraft (Gardner *et al*., 2000). Optimal selection and subsequent transfer of embryos with higher implantation potential may minimize the time to pregnancy (van Loendersloot *et al*., 2014). The limitations imposed by morphology-based embryo selection - short periods for which embryo development is assessed and reliance on the level of expertise of the attending embryologist - have turned time-lapse technology into a tempting embryo selection tool and added another dimension to traditional morphology classification grades (Desai *et al*., 2014). Time-lapse technology was recently introduced in the field of ART along with new incubators containing inverted microscopes and cameras, which allow embryos to be cultured uninterruptedly while their development is recorded. Undisturbed culture systems have been reported as safe (Cruz *et al*., 2012) and may potentially provide a culture environment leading to increased blastocyst formation (Cruz *et al*., 2012; Zhang*et al*., 2010), implantation, and clinical pregnancy rates (Cruz *et al*., 2012; Meseguer *et al*., 2012; Kirkegaard *et al*., 2012).

In addition to the benefits of uninterrupted culture, time-lapse systems provide for extensive data sets. Groups from all over the world are studying cell movement, division, and other events unexplored until now, such as reverse cleavage. Numerous studies have investigated the relationship between morphokinetics and embryo competence (Wong *et al*., 2010; Meseguer *et al*., 2011; Cruz *et al*., 2012; Dal Canto *et al*., 2012; Hashimoto *et al*., 2012). However, evidence of sophisticated time-lapse systems for morphokinetic algorithms that may predict successful outcome is limited (Kaser & Racowsky, 2014). The inability to effectively apply a published embryo selection model or algorithm in different settings is a recurring issue (Best *et al*., 2013; Adolfsson & Andershed, 2018) and suggests that validating the clinical use of time-lapse technology for embryo selection in each laboratory should start with the characterization of optimal growth patterns for human embryos within each individual *in vitro* culture system (Desai *et al*., 2014).

The aim of this study was to analyze whether there is agreement between morphological grading and morphokinetic parameters in blastocyst stage embryos cultured in a time-lapse system, which may be helpful to predict embryo potential at early developmental stages.

## MATERIAL AND METHODS

This retrospective cohort study was based on data collected from our clinic’s database. Patients undergoing ART treatment between October 2017 and May 2019 using a time-lapse system were included in this study.

Ovarian stimulation and oocyte retrieval were performed using standard protocols. All oocytes underwent intracytoplasmic sperm injection (ICSI) and placed individually in culture dishes (EmbryoSlide^®^, Vitrolife) with universal media (CSCM Complete- Irvine^®^) covered with 2 mL of mineral oil. The embryos were cultured in a time-lapse system (EmbryoScope Plus^®^, Vitrolife, Sweden) at 37ºC, 7.2% CO_2_ and 5% O_2_ until the blastocyst stage (day 5, 6, or 7). Depending on the treatment indication, the blastocysts were then either transferred into the uterus or frozen using standard vitrification techniques.

All embryos that achieved the blastocyst stage were analyzed using the time-lapse images recorded during uninterrupted culture. The EmbryoScope Plus^®^ system captured images from the embryos every 10 minutes in 11 different focal planes. On software EmbryoViewer^®^, the morphokinetic parameters were annotated by two experienced embryologists: time of pronuclei fading (tPNf), time to cleavage into two (t2), three (t3), four (t4), five (t5), and eight (t8) cells, time to start of blastulation (tB), the inner cell mass (ICM), and trophectoderm (TE) grade according to Gardner *et al*. (2000). Morphokinetic parameters were selected from the literature (Dal Canto *et al*., 2012).

The time to cleavage is defined as the first observed frame in which the newly formed blastomeres are completely separated by confluent cell membranes and tB is the time for which the embryo undergoes cavitation and the blastocoel is large enough to push the trophectoderm against the zona pellucida and the latter starts to grow thinner. Embryo cell quality has been described based on a two-letter classification system (e.g.: “AA”, “CB”), with the first letter grading the ICM and the second the TE. The descriptions for ICM quality are as follows: “A” for a tightly packed ICM with many cells; “B” for a loosely grouped ICM with several cells; and “C” for an ICM with very few cells. TE grading is as follows: “A” for a TE with many cells forming a cohesive epithelium; “B” for a TE with few cells forming a loose epithelium; and “C” for a TE with very few large cells (Alfarawati *et al*., 2011). Top-quality blastocysts are graded as AA, AB, BA, or BB.

Our study looked into 620 cycles. Cycles with donor eggs were not included. A total of 2085 blastocysts were analyzed. Mean maternal age was 37.04±3.61 years. Morphokinetic parameters and morphology grading were compared using one-way ANOVA followed by Tukey’s test (*p* values ≤ 0.05 were considered significant).

## RESULTS

**Figure 1:**
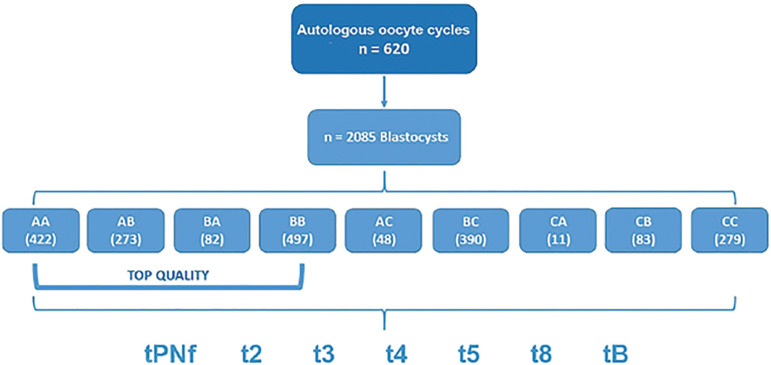
Study Design

Figure 1 illustrates the study design. Blastocysts were graded based on morphology as AA (n=422), AB (n=273), BA (n=82), BB (n=497), AC (n=48), BC (n=390), CA (n=11), CB (n=83), or CC (n=279).

Morphokinetic parameters at different times are described in Table 1. Morphokinetic parameters assigned significantly different morphology grades are summarized in Table 2. In the group of embryos assigned top grades, t8 was significantly different between AA (57.78±8.11 hours) and BB (60.36±9.78 hours); and tB was significantly different between AA (104.02±7.73) and AB (105.31±8.96) and between BA (103.57±8.27) and BB (109.44±9.40 hours).

**Table 1:** Morphokinetic parameters in hours according to morphology grading.

\	tPNf	t2	t3	t4	t5	t8	tB
** AA **	23.44±3.58	26.77±4.11	37.15±4.31	38.21±4.28	49.43±6.16	57.78±8.11	104.02±7.73
** AB **	23.38±2.89	26.51±3.16	36.92±4.45	38.36±4.40	48.99±6.10	58.64±8.82	105.31±8.96
** BA **	23.27±2.91	26.14±3.42	36.80±3.92	38.38±4.58	49.46±5.38	57.29±8.59	103.57±8.27
** BB **	24.07±3.25	27.05±3.66	37.55±4.29	39.10±4.77	49.91±7.25	60.36±9.78	109.44±9.40
AC	23.83±2.30	26.78±3.03	35.91±4.41	38.07±4.63	47.65±8.67	61.09±10.26	108.08±10.14
BC	23.89±3.12	27.22±3.96	36.36±5.26	38.53±5.58	47.70±8.40	61.01±11.46	112.08±11.50
CA	23.29±2.01	26.82±3.62	36.73±6.12	37.23±5.96	49.00±9.27	61.32±10.40	111.22±10.76
CB	23.73±2.80	27.46±4.36	36.76±4.83	38.85±4.47	48.41±7.04	60.03±10.04	112.33±10.66
CC	24.60±3.34	27.91±4.36	37.10±5.80	39.72±6.69	48.17±9.88	64.02±13.87	115.71±12.71

Values are expressed as Mean ± standard deviation.

**Table 2:** Significance of morphokinetic parameters according to morphology grading.

	AA	AB	BA	BB	AC	BC	CA	CB	CC
** AA **		\	\	t8**;tB***	\	t5*;t8***; tB***	\	tB***	tPNf*;t2**; t4**;t8**; tB***
** AB **	\		\	tB***	\	\	\	\	tPNf*;t2***; t4*;t8***; tB***
** BA **	\	\		tB***	\	tB***	\	tB***	t2** t8*** tB***
** BB **	\	\	\		\	t3** t5*** tB***	\	\	t8*** tB***
AC	\	\	\	\		\	\	\	tB***
BC	\	\	\	\	\		\	\	t8** tB***
CA	\	\	\	\	\	\		\	\
CB	\	tB***	\	\	\	\	\		\
CC	\	\	\	\	\	\	\	\	

* *p*≤0.05;

** *p*≤0.01;

*** *p*≤0.001.

Top quality blastocyst grades are in bold type.

When blastocysts assigned high and low grades were compared, differences were seen on tPNf between blastocysts graded AA (23.44±3.58 hours) and AB (23.38±2.89) relative to CC (24.60±3.34 hours); on t2 between blastocysts graded AA (26.77±4.11), AB (26.51±3.16) and BA (26.14±3.42 hours) relative to CC (27.91±4.36 hours); on t4 between blastocysts graded AA (38.21±4.28) and AB (38.36±4.40 hours) relative to CC (39.72±6.69 hours); on t8 between blastocysts graded AA (57.78±8.11), AB (58.64±8.82), BA (57.29±8.59) and BB (60.36±9.78 hours) relative to CC (64.02±13.87); and tB between blastocysts graded AA (104.02±7.73), AB (105.31±8.96), BA (103.57±8.27) and BB (109.44±9.40 hours) relative to CC (115.71±12.71 hours).

## DISCUSSION

Morphokinetics has gained new relevance in embryo selection with the introduction of time-lapse technology in IVF centers. Although other groups have explored the relationship between morphological characteristics and morphokinetic parameters, results have been less than consistent (Meseguer *et al*., 2011; Dal Canto *et al*., 2012). This paper looked into the association between time-lapse morphokinetic parameters and morphology-based blastocyst grading.

Our results showed that the time of pronuclei fading (tPNf), time to cleavage into two (t2), four (t4), and eight (t8) cells and time to start of blastulation (tB) were significantly different depending on blastocysts morphology grading. In early developmental stages, tPNf, t2, and t4 separated high (AA, AB, BA, BB) from low (CC) potential blastocysts. Time to cleavage into 8-cells separated blastocyst-stage embryos graded AA from blastocysts graded BB; and shorter time to blastulation correlated with higher quality embryos (AA, AB, BA).

In our practice, embryos are cultured to the blastocyst stage. Nevertheless, our culture and assessment protocols have changed since the introduction of time-lapse technology. Culture is uninterrupted with a single-step media and assessment is performed with the aid of the EmbrioViewer^®^ software using the images captured from multiple focal planes. The pictures are taken under low intensity red LED lighting (635 nm) and total exposure dose is much lower when compared to light exposure in protocols with one traditional manipulation step (Li *et al*., 2014). These changes improved the stability of the culture environment, a crucial factor for embryo blastulation, and may by itself increase the quality and total number of embryos in a patient cycle (Zhang *et al*., 2010). On the other hand, this system does not permit embryo rotation, which limits visual observation, in particular when blastomeres overlap or a high level of cytoplasmic fragmentation is present (Herrero & Meseguer, 2013).

The human zygote usually goes through its first cleavage early on day 2, between 24 and 27 hours after fertilization; it then cleaves to a 4- and 8-cell embryo on days 2 and 3, respectively, before compacting into a morula on day 4 and forming a blastocyst on days 5 or 6 (Nagy *et al*., 1994). When blastocyst formation initiates, the cells from the inner cell mass (ICM) and trophectoderm (TE) become more and more defined and visible as the fluid cavity enlarges - the blastocoel. At this stage, the embryologist categorizes and scores the embryos, and all attention is then diverted to deciding which embryo should be picked for transfer. The choice is usually made based on a quick analysis using a microscope and after the embryo has been categorized according to the Istanbul Consensus, with the one meeting the criteria more closely being transferred into the uterus. Regardless of whether the transfer is performed on days 2, 3, or 5, the decision is made harder when two or more embryos are available.

The first groups to use time-lapse technology in their protocols published studies concerning blastulation prediction. Wong *et al*. (2010) demonstrated the potential of time-lapse microscopy to predict blastocyst development with high sensitivity and specificity. An analysis of 100 embryos set out the standards for the duration of the first cytokinesis at 0-33 min; the time between the 2- and 3-cell stages at 7.8-14.3 hours; and the time between the 2- and 4-cell stages at 0-5.8 hours. However, there was no distinction in terms of blastocyst quality. Hashimoto *et al*. (2012) ran several experiments with donor eggs using a time-lapse imaging system based on individual embryo culture in poly(dimethylsiloxane) microwells monitored with a microscope inside the incubator. They found no differences in the time between pronuclei fading and first cell division when potentially high- and low-grading embryos were compared or among arrested embryos. Oocyte age did not affect the rates of development to the blastocyst stage either, but appropriate synchronization and timing of the second and third cleavages appeared to be critical to predict subsequent embryo development. In a search for published indicators of blastocyst formation and quality up to t8, Cetinkaya *et al*. (2015) found that cleavage synchronicity, specifically from 2 to 8 cells, was a better predictor when compared to absolute time-points. Despite their results, in which the authors indicated that such equation would be more likely to be transferrable between laboratories, time-point predictors can be more easily incorporated into protocol, particularly in IVF centers without time-lapse machines.

Meseguer *et al*. (2011) ran a retrospective study on cleavage times, blastomere size, and multinucleation from 247 transferred embryos with either failed or full implantation, and found that embryos with a t5 of 48.8 to 56.6 hours had a higher chance of developing into good-morphology blastocysts and higher implantation potential. Despite these findings, t5 was not significantly correlated with the development of good-morphology blastocysts in our study. Instead, the earlier parameters observed (tPNf, t2 and t4) distinguished poorer-quality (CC) from top-quality blastocysts (AA, AB, BA). Cruz *et al*. (2012) also reported that embryos with earlier cell division (significantly earlier from the 4-cell stage) developed into blastocysts with a cohesive TE and tightly packed ICM.

Considering top-quality blastocysts, tB was unexpectedly different for blastocysts assigned at least one A grade. AA, AB, and BA blastocysts reached tB after about 104 hours, whereas BB blastocysts reached tB after about 109 hours. Moreover, t8 was not only significantly longer in blastocysts graded CC (64.02 hours), but was also different when blastocysts graded AA and BB were compared (57.78 hours *vs.* 60.36 hours, respectively).

Since this study was designed to assess the relationship between morphology grading and morphokinetic parameters, the association of these parameters and clinical data such as pregnancy and live birth rates should be considered in future studies.

We highly recommend the implementation of time-lapse technology at fertility centers so that these parameters can be further validated and the technology used to its full potential.

## CONCLUSION

Our results showed that the first kinetic parameters (tPNf, t2, and t4) might be used to distinguish top-graded from low-graded blastocysts. Among top-graded blastocysts, t8 distinguished BB from AA blastocysts. Parameter tB can also be used to distinguish BB blastocysts from blastocysts graded as AA, AB, and BA. Time-point parameters may be used in IVF labs and fertility centers without time-lapse machines.
